# Comorbidity Structure of Psychological Disorders in the Online e-PASS Data as Predictors of Psychosocial Adjustment Measures: Psychological Distress, Adequate Social Support, Self-Confidence, Quality of Life, and Suicidal Ideation

**DOI:** 10.2196/jmir.3591

**Published:** 2014-10-28

**Authors:** Ali M AL-Asadi, Britt Klein, Denny Meyer

**Affiliations:** ^1^School of Health SciencesSwinburne University of TechnologyHawthornAustralia; ^2^Department of Arts and EducationGrande Prairie Regional CollegeGrande Prairie, ABCanada; ^3^DVC-Research & Innovation Portfolio; the School of Health Sciences and; the Collaborative Research NetworkFederation UniversityBallaratAustralia; ^4^National Institute of Mental Health ResearchThe Australian National UniversityCanberraAustralia; ^5^National eTherapy CentreSchool of Health SciencesSwinburne University of TechnologyHawthornAustralia

**Keywords:** comorbidity, comorbidity factors, comorbidity dimensions, structure of comorbidity, psychosocial adjustment, disorders, suicidal ideation, quality of life (QoL), self-confidence, distress, social support, online therapy, e-mental health, generalized anxiety disorder, obsessive-compulsive disorder, social anxiety disorder, posttraumatic stress disorder, PD, major depressive episode, anxiety disorders, insomnia, hypersomnia

## Abstract

**Background:**

A relative newcomer to the field of psychology, e-mental health has been gaining momentum and has been given considerable research attention. Although several aspects of e-mental health have been studied, 1 aspect has yet to receive attention: the structure of comorbidity of psychological disorders and their relationships with measures of psychosocial adjustment including suicidal ideation in online samples.

**Objective:**

This exploratory study attempted to identify the structure of comorbidity of 21 psychological disorders assessed by an automated online electronic psychological assessment screening system (e-PASS). The resulting comorbidity factor scores were then used to assess the association between comorbidity factor scores and measures of psychosocial adjustments (ie, psychological distress, suicidal ideation, adequate social support, self-confidence in dealing with mental health issues, and quality of life).

**Methods:**

A total of 13,414 participants were assessed using a complex online algorithm that resulted in primary and secondary Diagnostic and Statistical Manual of Mental Disorders (Fourth Edition, Text Revision) diagnoses for 21 psychological disorders on dimensional severity scales. The scores on these severity scales were used in a principal component analysis (PCA) and the resulting comorbidity factor scores were related to 4 measures of psychosocial adjustments.

**Results:**

A PCA based on 17 of the 21 psychological disorders resulted in a 4-factor model of comorbidity: anxiety-depression consisting of all anxiety disorders, major depressive episode (MDE), and insomnia; substance abuse consisting of alcohol and drug abuse and dependency; body image–eating consisting of eating disorders, body dysmorphic disorder, and obsessive-compulsive disorders; depression–sleep problems consisting of MDE, insomnia, and hypersomnia. All comorbidity factor scores were significantly associated with psychosocial measures of adjustment (*P*<.001). They were positively related to psychological distress and suicidal ideation, but negatively related to adequate social support, self-confidence, and quality of life.

**Conclusions:**

This exploratory study identified 4 comorbidity factors in the e-PASS data and these factor scores significantly predicted 5 psychosocial adjustment measures.

**Trial Registration:**

Australian and New Zealand Clinical Trials Registry ACTRN121611000704998; http://www.anzctr.org.au/trial_view.aspx?ID=336143 (Archived by WebCite at http://www.webcitation.org/618r3wvOG).

## Introduction

### Background

The co-occurrence of multiple psychological disorders (comorbidity) is a common and serious problem that needs to be better researched and understood. Kessler et al [1] found the lifetime prevalence of any disorder to be 46.4%, whereas 27.7% and 17.3% were the lifetime prevalences of 2 or more and 3 or more disorders, respectively. Kessler et al [2] found the 12-month prevalence of any disorder be 26.2%, whereas 5.8% met the criteria for 2 disorders and 6.0% met the criteria for more than 2 disorders. They also concluded that more than 40% of those who received 1 diagnosis also met the diagnostic criteria for a second diagnosis over a 12-month period [2]. Naturally, those suffering from multiple psychological disorders would more likely require greater assistance from their therapists should they seek help. Indeed, several studies concluded that comorbidity was consistently related to poor prognosis, higher rate of suicide, and high demand for professional help [3,4]. In addition, comorbidity has also been shown to be strongly related to symptom severity [2].

Although comorbidity has been studied extensively over the past 30 years, most studies on the structure of comorbidity have used 6-12 disorders at most and recommended that future research should include more disorders [5,6]. Furthermore, the use of dimensional rather than a discrete or dichotomous approach was suggested to solve the problems of generalizability and detection of the correlates of more severe mental disorders [7]. In addition, avoiding the reliance on threshold diagnoses in favor of consideration of subthreshold information has been recommended [8].

Krueger [7] argued for using a dimensional rather than a discrete/dichotomous approach and for including patients who met the criteria for multiple disorders when studying the structure of comorbidity because such patients were typical and more severely impaired. Consequently, Krueger argued that basing research designs on such samples would increase generalizability and the detection of correlates of more severe mental disorders. To this end, Krueger performed factor analysis on 10 mental disorders based on the *Diagnostic and Statistical Manual of Mental Disorders* (Third Edition, Revised; *DSM-III-R*) using the National Comorbidity Survey (NCS) sample of 8098 (collected between 1990 and 1992) and found 2 factors: an externalizing factor which included alcohol and drug dependency and antisocial personality disorder and an internalizing factor which was broken down into an anxious-misery factor, including depression, dysthymia, generalized anxiety disorder (GAD); and a fear factor, including social anxiety disorder (SAD), simple/specific phobia, agoraphobia, and panic disorder (PD). This model was criticized for the restricted range of 10 disorders, the reliance on lifetime diagnoses, and the exclusive reliance on threshold diagnoses without consideration of the subthreshold diagnostic information [8]. However, the structure of Krueger’s factors was later supported by several studies [2,6,9-12].

To review the literature on the comorbidity and the structure of comorbidity of all 21 disorders will require a great deal of space and is beyond the scope of this report. Therefore, we shall limit this summary review to disorders that have received the most attention and are the most prevalent: anxiety disorders, major depressive disorder (MDD), eating disorders, and alcohol and drug abuse and dependency disorders.

### Anxiety and Major Depressive Disorders

Comorbidity of anxiety disorders and MDD is among the most prevalent comorbid psychiatric conditions [13,14]. Estimates of comorbid anxiety disorders and MDD range from 15.9% to 61.9% in children and adolescents [15] and from 14.5% [16] to 57% [17-20] in specific populations of adults. In addition, sleep problems such as insomnia and hypersomnia have been consistently found to be associated with anxiety and mood disorders [21-25]. These studies suggest the existence of an anxiety-depression-insomnia comorbidity factor.

The co-occurrence of MDD and anxiety disorders has many clinical implications. Several studies found outpatients with MDD and at least 1 co-occurring anxiety disorder were significantly more likely to suffer from insomnia [21], personality disorders [26], more severe and chronic depression [16,27], and engage in suicidal ideation [18] and suicidal behavior [28] than individuals with only a MDD diagnosis.

GAD is highly correlated with mood disorders and has slightly lower correlations with anxiety disorders [29]. The tendency for GAD to be highly comorbid with other anxiety and depressive disorders has led some researchers to question its discriminant validity among diagnostic categories [30]. Researchers have pointed out that some of the core features of GAD are usually present in varying degrees in all anxiety disorders as well as mood disorders, detracting from the overall discriminant validity and reliability of GAD as a principal diagnosis. Factor analysis has also revealed a closer link between GAD and depressive disorders such as major depression, dysthymia, and major depressive episode (MDE) (the internalizing anxious-misery dimension) than between GAD and anxiety disorders such as social phobia, simple/specific phobia, PD, agoraphobia, and obsessive-compulsive disorder (OCD)—the internalizing fear dimension [5,6]. Some researchers suggested that GAD would be better categorized with the mood disorders whereas others suggested a complete revision of the hierarchical structure of the *DSM-IV* in which all anxiety and mood disorders would be grouped together and then partitioned into 3 subclasses with MDD, GAD, dysthymic disorder, and posttraumatic stress disorder (PTSD) forming a subclass of distress disorders [31-33].

Conversely, a more recent longitudinal study supported the *DSM-IV-TR* classification of GAD as an anxiety disorder. In an effort to determine whether GAD would be better classified as a mood disorder rather than an anxiety disorder, Beesdo et al [34] examined the risk patterns, incidence, developmental features, and comorbidity of anxiety and depressive disorders in 3021 individuals in a prospective longitudinal study conducted over a period of more than 10 years. They concluded that GAD was linked more closely to anxiety disorders than mood disorders in terms of risk associations in familial, temperamental, personality, and environmental variables. Moreover, temporal comorbidity of GAD showed a strong association between GAD and other anxiety disorders.

One of the earliest reviews of the literature on comorbidity among anxiety disorders is the one conducted by Brown and Barlow [30]. They highlighted several points: diagnoses of PD with or without agoraphobia and GAD were associated with some of the highest comorbidity rates between psychiatric conditions, core features of PD with or without agoraphobia and GAD were shared to some extent with all anxiety disorders, and substance abuse followed by GAD were the most commonly comorbid lifetime and current disorders experienced by war veterans with or without PTSD. They also concluded that, as with GAD, the discriminant validity of PD with or without agoraphobia was questionable because some of the central features in PD with or without agoraphobia (eg, anxious apprehension) were present in varying degrees in all anxiety and mood disorders. Subsequent studies found essentially the same associations and comorbidities—PD frequently co-occurs with other anxiety disorders [35-37].

### Depression, Anxiety, and Eating Disorders

Depressive and anxiety disorders are also frequently reported in those diagnosed with eating disorders. The lifetime prevalence of major depression ranges from 50% to 71% in anorexia nervosa and 50% to 65% in bulimia nervosa [38-41]. When the age was restricted to 12-18 years, 60% of adolescent girls with anorexia nervosa reported comorbid mood disorder [42]. On the other hand, when the age was broadened to include persons aged 11-68 years, 92% of the large female sample with anorexia nervosa had unipolar depression [43].

Although studies investigating the relationship between eating and anxiety disorders have produced somewhat mixed results [44-46], several researchers have found high rates of comorbidity between eating and anxiety disorders [43,47-50]. The few studies that employed control groups found significant comorbidity between anxiety disorders and anorexia nervosa and bulimia nervosa populations in comparison to non-eating disordered controls [46,51-54].

Moreover, Allen and Hollander [55] and Cororve and Gleaves [56] pointed out the significant overlap between the features of eating disorders (particularly anorexia nervosa) and body dysmorphic disorder (BDD). In both cases, body image distortion, excessive concerns and unreasonable preoccupation about physical appearance, and stressful dissatisfaction with one’s body are common features. Moreover, similar to the behaviors of individuals with OCD, excessive concerns and preoccupation over one’s body lead to anxiety-provoking obsessive thoughts about one’s self-image which might force the individual to engage in ritualistic-like behaviors to reduce the generated anxiety [55,57,58]. Therefore, all these studies suggest the existence of an eating-anxiety comorbidity factor and/or a body image-eating disorder comorbidity factor.

### Anxiety and Substance Abuse and Dependency Disorders

The relationship between psychological disorders and the use of drugs and alcohol has long been established [59-61]. McGovern et al [62] found substance use disorders were present in 42%, 27%, and 20% of patients diagnosed with mood disorders, anxiety disorders, and antisocial personality disorder, respectively. Krueger [7] found alcohol and drug dependence and antisocial personality disorder to make up the externalizing factor. Katz et al [63] performed confirmatory factor analysis on the same NCS sample used by Krueger [7], but this time with the inclusion of alcohol and drug abuse and dependence. They found alcohol and drug abuse to have strong negative loadings, whereas alcohol and drug dependency were found to have strong positive loadings on the externalizing factor. In addition, they found alcohol and drug abuse to have substantial loadings on the anxious-misery subfactor of the internalizing factor. They concluded that there appears to be a group of individuals with alcohol and drug abuse that is distinct in terms of comorbidity patterns and etiology from those with alcohol and drug dependency and from those with anxious-misery disorders (MDE, dysthymia, GAD). There appears to be some support, at least as far as alcohol is concerned, for the distinction between alcohol abusers and alcohol dependents in that, unlike abusers, alcohol dependency consists of a single latent dimension whereas alcohol abuse consists of many underlying latent dimensions [64]. These studies suggest the presence of a substance dependence comorbidity factor.

In summary, it appears that comorbidity of psychological disorders is a prevalent phenomenon with almost half of those receiving a diagnosis also receiving multiple diagnoses. Comorbidity is associated with more demands for services, poorer prognosis, higher rate of suicide, greater levels of severity, and poorer adjustment. Most studies on the structure of comorbidity focused on a few psychological disorders. Anxiety disorders, MDD, and eating disorders are the most widely researched. Results show that comorbidity of anxiety and MDDs are most prevalent. MDD was comorbid with GAD, OCD, PTSD, PD with or without agoraphobia, SAD, simple/specific phobia, insomnia, hypersomnia, and alcohol and drug abuse disorders. Anxiety disorders are particularly comorbid with GAD as well as with one another. In addition, significant comorbidity has been found between anxiety, depression, and eating disorders. Finally, although alcohol and drug abuse disorders appear to be comorbid with MDE and some anxiety disorders, alcohol and drug dependency disorders form a separate group with antisocial personality disorder.

The Mental Health Online (previously Anxiety Online) electronic psychological assessment screening system (e-PASS; an online psychological assessment screening system assessing for 21 *DSM-IV-TR* disorders) dataset (see [65,66] for further information), contains information on more than 13,000 individuals who have received 21 single and/or multiple psychological diagnoses based on dimensional measures referred to as clinical disorder severity scales. These elements of the e-PASS dataset provide a unique opportunity to examine the structure of comorbidity of 21 psychological disorders based on dimensional scales that reflect information that covers subthreshold to postthreshold diagnoses.

**Figure 1:**
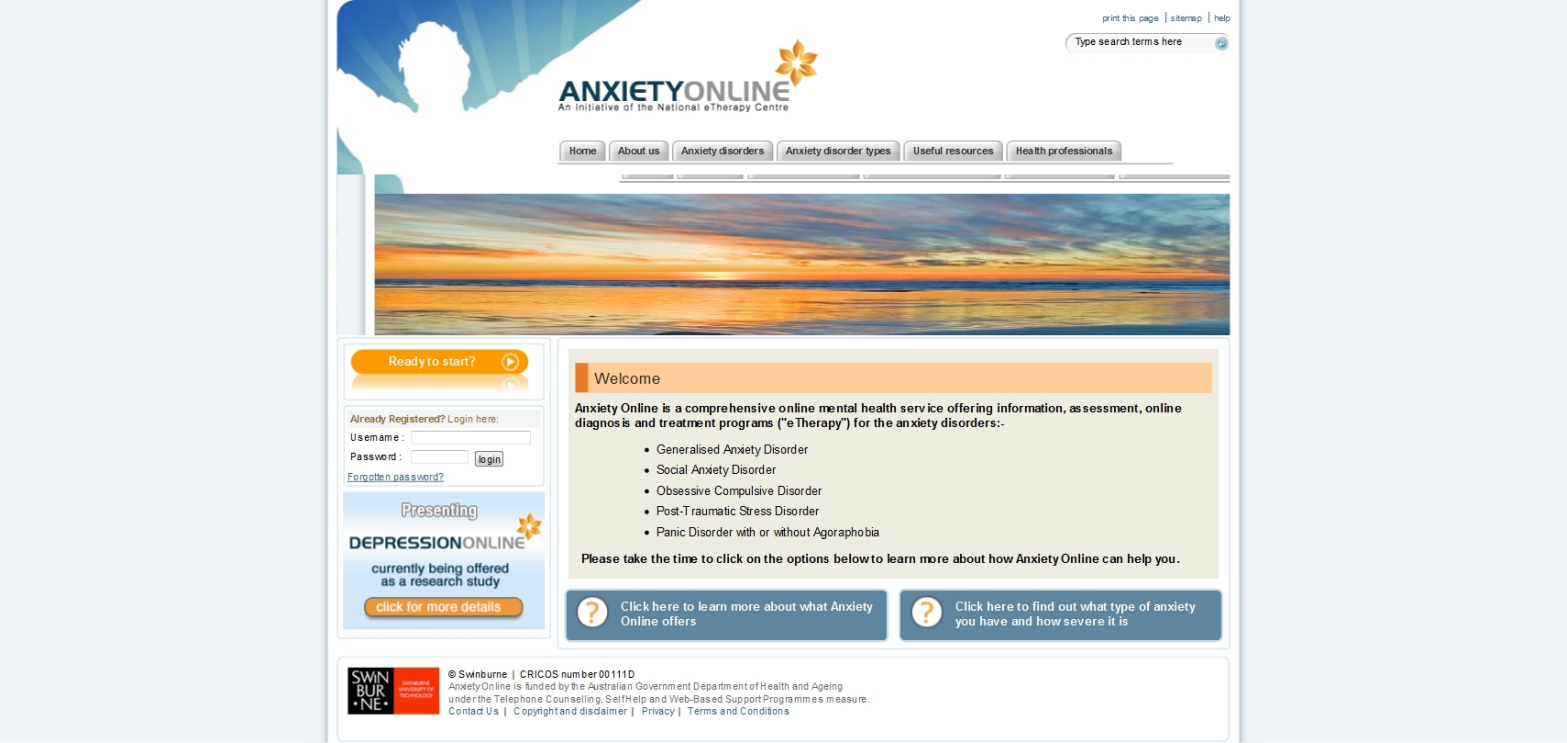
Screenshot of Anxiety Online home page (now Mental Health Online).

The purpose of this exploratory study was 2-fold. Firstly, we used the dimensional severity scales of the e-PASS data to explore the underlying structural dimensions of comorbidity (henceforth, comorbidity factors) of the 21 psychological disorders diagnosed by the e-PASS part of the Mental Health Online platform (Figure 1) [67]. Secondly, we validated the resulting comorbidity factors by determining their relative performance for predicting psychological distress as measured by Kessler-6 total score, suicidal ideation, quality of life, level of self-confidence, and adequate social support. We predicted that comorbidity factor scores would be positively related to suicidal ideation and Kessler-6 total score, but negatively related to quality of life, self-confidence, and adequate social support.

For those diagnosed with more than 1 disorder, it has been found that the number of diagnosed psychological disorders is positively related to the level of psychological distress [68] and to suicidal ideation [3,4], whereas negatively related to patients’ level of self-confidence, quality of life [69,70], and social support [71,72]. Therefore, any comorbidity factor scores should adequately predict these validation variables.

## Methods

### Procedure

The Mental Health Online platform consists of 4 centers: psychoeducational information, psychological screening and assessment, online treatment, and health care practitioner training. The psychoeducational center is a website that provides psychoeducational information. The assessment center contains the e-PASS. The treatment center provides and manages the 5 anxiety treatment programs and the more recent depression, bulimia nervosa, and insomnia treatment programs. The training center provides the eTherapist training programs and the health care practitioner portal. The online psychological assessment and referral program, e-PASS, includes a variety of demographic and personal questions, the Kessler-6 [73], and the Suicidal Warnings (ideation) measure, as well as the online diagnostic program. Individuals can access the Mental Health Online service from anywhere in the world provided they have an Internet connection. People can complete e-PASS if they are interested in the psychological assessment function and/or if they are interested in online treatment. Those who want to undertake the e-PASS are first required to register and consent to the Mental Health Online terms and conditions [67]. The procedures for collecting and reporting the Anxiety Online data were approved by the Swinburne University Human Research Ethics Committee.

### Diagnostic Assessment

Based on an individual’s response to some of the e-PASS questions, a person may be given a primary diagnosis and/or multiple secondary diagnoses. Primary or secondary diagnosis is determined by the reported presence of *DSM-IV-TR* symptoms and the average score on severity scales, each of which assesses the level of distress and interference caused by the symptoms of a particular disorder. A total of 21 clinical disorders are assessed by the e-PASS (see [65,66] for more details).

The disorder-specific severity score is the average of the scores on 6 questions that assess how distressed and how many of the symptoms of a given disorder interfere in one’s life. For example, an individual who meets the diagnostic symptomatology of PD has to rate each of the following questions on a scale from 0 indicating no distress or interference to 8 indicating extremely severe distress or interference as indicated subsequently:

Currently, how bothered or distressed are you by your panic attack(s) and or worries about having another panic attack, and or any changes in your behavior/routine because of these panic attacks, in each of the following areas of your life:

1. Everyday living (eg, shopping, cleaning the house, preparing food)
2. Social (eg, meeting friends or family, forming/maintaining relationships)
3. Work or study (eg, attending work or classes, performing tasks)

Currently, how much interference do your panic attack(s), and or worries about having another panic attack, and or changes in your behavior/routine because of these panic attacks cause, in each of the following areas of your life:

1. Everyday living (eg, shopping, cleaning the house, preparing food)
2. Social (eg, meeting friends or family, forming/maintaining relationships)
3. Work or study (eg, attending work or classes, performing tasks)

A person who does not endorse the initial *DSM-IV-TR* symptom criteria questions for a particular disorder is not presented with the questions assessing their level of distress and interference of those symptoms and is assigned a severity score of zero. Those who do endorse the initial *DSM-IV* symptom criteria questions for a particular disorder are presented with the 6 distress and interference questions allowing the calculation of a mean severity score ranging from 0 to 8. A mean distress and interference severity score of 3.5 or greater is considered sufficient to warrant a clinical diagnosis. Those whose mean distress and interference severity scores are less than 3.5 are considered to warrant a subclinical (or subthreshold) diagnosis.

The e-PASS diagnostic system was modeled after and informed by the Anxiety Disorders Interview Schedule (ADIS) clinician rating scale (Likert scale: 0=no symptoms, 4=mild presence of the disorder, 8=very severe presence of the disorder). Most “total scores” would not be a whole number because the system used 6 rating scales and then averaged them. Consequently, the final scores were rounded up or down to the nearest whole number. Thus, a 4 is the typical score by a clinician using the ADIS that indicates the “presence” of a disorder. However, considering the decimal places resulting from the e-PASS averaging of the 6 rating scales, those scoring 3.5 or more were deemed clinical.

The psychometric properties of the e-PASS measures were shown to have high test-retest reliability and reasonable convergent validity with the structured clinical interviews (D Nguyen, unpublished PhD thesis, Swinburne University, 2013). However, the small sample size and some disagreement with the structured clinical interviews in terms of the severity levels required for a clinical diagnosis suggest that further validation studies with large sample sizes are needed.

For the purpose of this work, we did not use the *DSM-IV-TR* criteria for clinical diagnosis. Instead, we used the previously described severity score without consideration of the level of severity that would warrant clinical diagnosis. The average severity scores provide the opportunity to examine the structure of comorbidity of 21 psychological disorders based on a dimensional scale that reflects information that covers subthreshold to postthreshold diagnoses. This dimensional approach should solve the problems of generalizability and detection of the correlates of more severe mental disorders as suggested by Krueger [7]. Also, the inclusion of subthreshold symptomatology should avoid the risk of finding artifactual explanations as was suggested by Wittchen et al [8].

### Participants

A total of 13,414 individuals completed the e-PASS phase between October 2009 and October 2012 and received at least 1 clinical diagnosis. The sample consisted of 3974 (29.63%) males whose age ranged between 18-85 years with a mean of 36.88 (SD 12.59) years, and 9440 (70.37%) females whose ages ranged between 18-86 years with a mean of 33.66 (SD 11.57) years. A total of 749 (5.58%) participants received a clinical diagnosis (severity score greater than 3.5) of only 1 disorder, leaving 12,665 (94.42%) participants who were classified as having a clinical or subclinical diagnosis for 2 or more of the 21 disorders assessed by e-PASS.

In this study, the score that each participant received before treatment on the following variables were used to validate the resulting comorbidity factors: Kessler-6 total score, suicidal ideation, adequate social support (“Do you feel you have adequate level of social support or engagement in social and/or community activities?”), self-confidence (“Please rate your overall level of self-confidence when it comes to managing your own mental health”), and quality of life (“Please rate your overall quality of life”). The suicidal ideation and adequate social support measures were based on binary yes/no answer responses. The level of self-confidence and the quality of life measures were based on a Likert scale from “very poor” to “very good.” For the purpose of this study, the last 2 measures were converted to binary scale, with all those who gave a rating of very poor and poor coded as zero and those who gave a rating for good and very good coded as “1”.

### Analysis

A series of principal component analyses were conducted to develop new comorbidity factors from the severity scores described previously. The descriptive statistics are reported for the 21 severity scores and for the validation variables used to validate the new comorbidity factors.

The pervasive nature of depression and anxiety disorders meant that a typical factor analysis was not appropriate for the extraction of the comorbidity factors. In particular, it was not appropriate to search for a simple structure for the loadings of the severity scores on the final comorbidity factors. Instead, the goal was to derive the underlying dimensions that explained as much of the variation in the severity scores as possible while recognizing the importance of depression and anxiety in the majority of the comorbidity factors. The relative accuracy of the severity score measures suggested that a principal component analysis (PCA) should be used to construct the comorbidity factors, rather than factor analysis with principal axis factoring or maximum likelihood extraction of factors. This choice was supported when comparative analyses performed with maximum likelihood and principal axis factoring extraction methods failed to produce results supported in the literature.

Monte Carlo simulation (parallel analysis) [74] was carried out to determine the optimum number of components at each step, based on the work of O’Connor [75]. Initially an oblimin rotation was applied to the factors, allowing correlation between the resulting comorbidity factors, but when these correlations were found to be very weak, the oblimin rotation was replaced by a varimax rotation ensuring that the resulting comorbidity factors were independent of each other. At each step of the analysis, disorders that loaded weakly on all factors were removed.

In the second part of the analysis, comorbidity factor scores were validated using linear and logistic multiple regression to test their relationships with psychological distress (as measured by the Kessler-6), suicidal ideation, adequate social support, self-confidence, and quality of life. All analyses were carried out using SPSS version 20 (IBM Corp, Armonk, NY, USA).

## Results

### Principal Component Analysis

The number of clinical diagnoses among those who received more than 1 diagnosis ranged from 1 to 16 with a mean of 5.07 (SD 2.41). The number of diagnoses was significantly and positively correlated with the psychological distress Kessler-6 total score (*r*=.54, *P*<.001), negatively with quality of life (*r*=–.32, *P*<.001), negatively with self-confidence (*r*=–.29, *P*<.001), and negatively with level of adequate social support (*r*=–.24, *P*<.001). The 21 disorders and all descriptive statistics are shown in Table 1. The validation variables and their descriptive statistics are shown in Table 2.

**Table 1:** The frequency of severity of 21 psychological disorders.

e-PASS Assessment			Severity of disorder (N=13,414)		Mean (SD)
			Clinical	Subclinical	
**Psychological disorders, n (%)**					
	Panic disorder (PD) with or without agoraphobia		2970 (22.14)	1809 (13.49)	1.43 (2.21)
	Agoraphobia without a history of PD		1107 (8.25)	1447 (10.79)	0.61 (1.47)
	Social anxiety disorder (SAD)		3504 (26.12)	4031 (30.05)	1.92 (2.20)
	Specific phobia		1823 (13.59)	3055 (22.77)	1.10 (1.86)
	Generalized anxiety disorder (GAD)		4464 (33.28)	4366 (32.55)	2.35 (2.28)
	Obsessive-compulsive disorder (OCD)		1033 (7.70)	2730 (20.35)	0.74 (1.52)
	Posttraumatic stress disorder (PTSD)		1461 (10.89)	2974 (22.17)	0.96 (1.74)
	Major depressive episode (MDE)		5131 (38.25)	4188 (31.22)	2.63 (2.34)
	Anorexia nervosa		41 (0.31)	64 (0.48)	0.02 (0.31)
	Bulimia nervosa		804 (5.99)	931 (6.94)	0.46 (1.46)
	Binge eating disorder		316 (2.36)	682 (5.08)	0.21 (0.89)
	**Substance dependence**				
		Cannabis	127 (0.95)	839 (6.25)	0.14 (0,63)
		Stimulants	48 (0.36)	492 (3.67)	0.07 (0.44)
		Opioids	29 (0.22)	313 (2.33)	0.05 (0.34)
		Sedatives	104 (0.78)	1218 (9.08)	0.18 (0.65)
		Alcohol dependence	321 (2.39)	2201 (16.41)	0.38 (0.96)
	Somatization disorder		179 (1.33)	51 (0.38)	0.09 (0.70)
	Body dysmorphic disorder		1503 (11.20)	649 (4.84)	0.71 (1.79)
	Problem/pathological gambling		49 (0.37)	457 (3.41)	0.07 (0.42)
	Insomnia		4049 (30.18)	4546 (33.89)	2.20 (2.23)
	Hypersomnia		1060 (7.90)	827 (6.17)	0.53 (1.50)

**Table 2:** The frequency of and descriptive statistics of the validation variables.

Validation variables	Frequency (N=13,414)	Mean (SD)
Suicidal ideation (yes), n (%)	5079 (3.86)	
Quality of life (high), n (%)	6877 (51.27)	
Adequate social support (high), n (%)	6024 (44.91)	
Self-confidence (high), n (%)	3888 (28.98)	
Pre-Kessler-6 (total score)		17.49 (4.90)

All but 6 of 210 severity score correlations among disorder pairs were positive, and all but 11 of 210 correlations were statistically significant (*P*<.05). The Kaiser-Meyer-Olkin (KMO) of 0.80 confirmed that there was sufficient correlation in the data to warrant a PCA analysis of this nature.

To determine the optimum number of comorbidity factors, both methods of Monte Carlo simulation, random data and permutation, indicated the presence of 4 underlying dimensions, as suggested by the literature review. Disorders that loaded weakly (<.3) on all factors were removed which resulted in the removal of problem/pathological gambling, somatization disorder, and binge eating disorder because none of these disorders featured prominently in the comorbidity literature. The resulting PCA model accounted for 42.9% of the total variance in the remaining 18 severity scores when an oblimin rotation was used. However, the component correlation matrix showed all correlations to be less than .3 in absolute value, suggesting that a simpler solution with 4 distinct comorbidity factors was possible with a varimax rotation.

The penultimate 4-component model indicated that the fourth component consisted of a negative loading of PD with or without agoraphobia (–.59) and a positive loading of agoraphobia without a history of PD (.86). This was a reflection of the mutual exclusivity of these 2 classifications. That is, according to *DSM-IV-TR*, a person can only be diagnosed with 1 or the other but not both of these disorders at the same time. Also, although a KMO of 0.8 was indicative of the suitability of the dataset for factor analysis, examination of the anti-image of the correlation matrix revealed that the measures of sampling adequacy for each individual variable ranged from 0.69 to 0.91 except for the agoraphobia without a history of PD, which was rather poor at 0.4 suggesting that this variable should be removed. For these reasons, it was decided to remove agoraphobia without a history of PD from the analysis and develop a new model.

After removing agoraphobia without a history of PD, the KMO value increased to 0.85 and a new 4-component model emerged accounting for 43.9% of the variance in severity scores. As shown in Table 3, the final 4-component model based on a varimax rotation provided support for the 4 comorbidity factors suggested by the literature. The first component included anxiety disorders (specific phobia, PD with or without agoraphobia, GAD, SAD, PTSD, and OCD) with MDE and insomnia having the highest loading on this anxiety-depression factor. The second component showed moderate loadings for alcohol and other drug dependency–related disorders (stimulant dependence, cannabis dependence, opioid dependence, alcohol dependence, and sedative dependence) confirming the existence of a substance abuse comorbidity factor. The third component exhibited high to moderate positive loadings for bulimia nervosa, BDD, anorexia nervosa, and OCD, confirming the presence of a body image–eating factor. The fourth component showed high to moderate positive loadings for MDE, insomnia, and hypersomnia, confirming the existence of a depression-insomnia comorbidity factor.

**Table 3:** Component weights for principal component analysis with varimax rotation.

Disorder severity	Component			
	Anxiety-depression	Substance abuse	Body image–eating	Depression–sleep problems
Specific phobia	.715			
PD with or without agoraphobia	.695			
GAD	.654			
SAD	.622			
MDE	.541			.509
PTSD	.537			
Insomnia	.481			.454
OCD	.424		.356	
Stimulant dependence		.638		
Cannabis dependence		.596		
Opioid dependence		.513		
Sedative dependence		.499		
Alcohol dependence		.493		
Bulimia nervosa			.791	
BDD			.705	
Anorexia nervosa			.408	
Hypersomnia				.745

### Comorbidity Factor Scores

Principal component scores representing these 4 comorbidity factors were calculated for each participant. The first component represents the anxiety-depression comorbidity factor. The second component represents the substance abuse comorbidity factor. The third component represents the body image–eating comorbidity factor. The fourth component represents the depression–sleep problems comorbidity factor.

The 4 comorbidity factor scores were then tested for their relationships with the validation measures. These uncorrelated factor scores served as the independent variables for multiple regression analysis with the Kessler-6 total score serving as the dependent variable. The results are shown in Table 4. The regression coefficients for the 4 comorbidity scores were positive and statistically significant (*P*<.001), explaining 44% of the variation in the Kessler-6 total score.

**Table 4:** Results of regression analysis for the comorbidity factor scores and Kessler-6 total score

Comorbidity factors	B	SE	*t*_1_	*P*	*R*	*R* ^*2*^
					.66	.44
Anxiety-depression	2.49	.03	78.4	.001		
Substance abuse	0.43	.03	13.6	.001		
Body image–eating	0.97	.03	30.5	.001		
Depression–sleep problems	1.81	.03	57.1	.001		

In addition, these comorbidity factor scores were related to 4 other variables: suicidal ideation, adequate social support, self-confidence, and quality of life using binary logistic regression as shown in Table 5. The final binary logistic regressions with forward selection resulted in significant odds ratios for all 4 comorbidity factor scores. On average, increased odds for experiencing suicidal ideation of 108%, 36%, 48%, and 91%; for inadequate social support of 71%, 10%, 16%, and 49%; for having low self-confidence of 30%, 13%, 52%, and 64%; and for poor quality of life of 31%, 22%, 33%, and 81% for each additional point scored on anxiety-depression, substance abuse, body image–eating, and depression–sleep problems factors, respectively.

**Table 5:** Binary logistic regression for suicidal ideation, inadequate social support, low self-confidence, and poor quality of life in relation to comorbidity factor scores.

Comorbidity factors		Wald (*df*=1)	*P*	OR (95% CI)
**Suicidal ideation**				
	Anxiety-depression	1139.11	.001	2.08 (1.99-2.17)
	Substance abuse	177.48	.001	1.36 (1.30-1.42)
	Body image–eating	338.30	.001	1.48 (1.42-1.54)
	Depression–sleep problems	886.34	.001	1.91 (1.83-2.00)
	Constant	688.91	.001	0.59
**Inadequate social support**				
	Anxiety-depression	650.90	.001	1.71 (1.64-1.78)
	Substance abuse	21.65	.001	1.10 (1.06-1.14)
	Body image–eating	55.50	.001	1.16 (1.11-1.20)
	Depression–sleep problems	377.74	.001	1.49 (1.43-1.55)
	Constant	183.94	.001	1.28
**Low self-confidence**				
	Anxiety-depression	805.52	.001	2.30 (2.17-2.44)
	Substance abuse	23.41	.001	1.13 (1.08-1.19)
	Body image–eating	198.49	.001	1.52 (1.43-1.61)
	Depression–sleep problems	335.03	.001	1.64 (1.56-1.73)
	Constant	2287.57	.001	3.07
**Poor quality of life**				
	Anxiety-depression	1267.24	.001	2.31 (2.20-2.42)
	Substance abuse	77.36	.001	1.22 (1.17-1.27)
	Body image–eating	174.99	.001	1.33 (1.27-1.38)
	Depression–sleep problems	710.34	.001	1.81 (1.74-1.89)
	Constant	0.66	.42	1.02

These results confirm that the 4 comorbidity factor scores are associated with the validation variables that are commonly associated with psychological distress, thereby providing some validation for these underlying dimensions of comorbidity.

## Discussion

### Initial Structure

Using the scores on dimensional scales measuring the severity of 21 psychological disorders, the underlying structure of comorbidity of these disorders was examined using the method of PCA. Somatization disorder, pathological gambling, and binge eating disorder were systematically removed from analysis because of weak loadings of less than 0.3 on all components. The penultimate model consisted of 4 components, the last of which consisted of only a negative loading for PD with or without agoraphobia and a positive loading for agoraphobia without a history of PD, reflecting the mutual exclusivity of these 2 diagnoses. According to *DSM-IV-TR*, a person can only be diagnosed with 1 or the other but not both disorders at the same time. This also suggests that there are differences between the 2 groups even though the 2 groups share common etiological factors. This finding is consistent with the finding of Katz et al [63], but is contrary to the new *DSM-5* criterion that no longer allows agoraphobia without PD. However, Katz et al [63] found a negative relationship between PD with or without agoraphobia and agoraphobia without a history of PD, but Krueger [7] found a positive relationship between these 2 disorders. In view of these previous findings and the high component loadings for only these 2 disorders on our fourth component, we removed agoraphobia without a history of PD producing a new factor model containing 17 disorders loading on 4 reconfigured components.

### Comorbidity Structure

The underlying dimensional structure of the 17 psychological disorders consisted of 4 factors that accounted for 43.9% of the total variance. The first comorbidity factor, labeled anxiety-depression, consisted of all anxiety disorders (specific phobia, PD with or without agoraphobia, GAD, SAD, PTSD, and OCD) with MDE and insomnia. The second comorbidity factor, labeled substance abuse, consisted of alcohol and substance dependency disorders (alcohol dependence, cannabis dependence, stimulant dependence, opioid dependence, and sedative dependence). The third comorbidity factor, labeled body image–eating, consisted of body image and eating problems (bulimia nervosa, BDD, anorexia nervosa, and OCD). The fourth comorbidity factor, labeled depression–sleep problems consisted of MDE, insomnia, and hypersomnia. It is important to note that these comorbidity factors are unlikely to be a reflection of the inability of these dimensional severity scales to adequately distinguish between the different disorders. It is the lack of correlation between these 4 factors that suggests that these clusters of disorders are distinct rather than the severity scales lacking the specificity to distinguish between these disorders.

We should also emphasize here that we are not aware of any work on comorbidity or the structure of comorbidity of psychological disorders in the context of online programs. Therefore, this work may be the first in addressing this area. Consequently, we are comparing the results of this study with results based on using in-clinic samples. Although there is no logical reason to expect the comorbidity of psychological disorders to be different for an online population from an in-clinic population, caution is warranted.

These results are generally supportive of Krueger’s model [7]. The substance abuse factor corresponds with Krueger’s externalizing factor minus the antisocial personality disorder (not included in our data). The fact that all alcohol and drug dependency classifications are highly loaded on 1 factor implies that they may be largely determined by a single latent dimension. This finding is consistent with the work of Katz et al [63] who suggested that alcohol and drug dependency have a common latent dimension and the work of Slade et al [64] who concluded that alcohol dependency consisted of 1 latent dimension.

The anxiety-depression factor corresponds with Krueger’s internalizing factor [7]. However, our results do not support the separation of the internalizing factor into anxious-misery (depression, dysthymia, GAD) and fear (SAD, specific phobia, agoraphobia, PD) subfactors [7]. This could be due to the differences between our dataset and Krueger’s dataset [7]. Our results are based on 17 disordered classifications that were assessed on dimensional scales that included subthreshold scores whereas Krueger’s data [7] were based on 10 disorders that were assessed on scales that relied exclusively on threshold diagnoses.

It has long been known that anxiety disorders tend to be comorbid with depression as well as each other. Our results are consistent with this notion that anxiety disorders and depression share many common symptoms. The inclusion of insomnia in this group of disorders is understandable because it is often the case that people who are suffering from insomnia are also suffering from 1 or more anxiety and/or mood disorders [21,22]. This assertion becomes even clearer when we consider the depression-sleep problem factor. Insomnia and hypersomnia are sometimes associated with increases in anxiety and depression [23-25]. Although insomnia or hypersomnia could be symptoms of anxiety or mood disorders, they could also lead to the experience of anxiety and/or depressive symptoms.

The body image-eating factor consists of disorders that are related to the perception of body image. People suffering from these disorders are not satisfied with the way they look, but each group deals with this dissatisfaction differently. The inclusion of OCD with these disorders is understandable and was predicted. Sufferers of eating disorders and body dysmorphic disorder share excessive concerns and preoccupation about physical appearance coupled with dissatisfaction with their bodies that commonly lead them to develop a distorted self-image. The anxiety-provoking obsessive thoughts about their appearance force them to engage in ritualistic behaviors to reduce their anxiety. This pattern of anxiety-provoking obsessive thoughts reduced by ritualistic-like behaviors is typical of OCD sufferers. Those with eating disorders obsess over their body weight and engage in behaviors that reduce their weight, such as limiting food intake, vigorous exercises, and using laxatives and/or forced purging. Those with BDD obsess over their appearance and engage in behaviors that reassure them, such as frequently checking with others in mirrors, comparing themselves to others, camouflaging themselves, and seeking cosmetic surgeries. These results are consistent with findings of Allen and Hollander [55] and Cororve and Gleaves [56], and with the finding of Rauch et al [57] who indicated that the same brain structures were implicated in BDD and OCD, and Phillips [58] who suggested that treatment for OCD is useful for BDD. However, it should be noted that, aside from OCD, our results do not support the existence of an eating-anxiety comorbidity factor.

Finally, now that the *DSM-5* has been introduced, there will be a need to revise the e-PASS assessment tool based on the new criteria. This revision will likely be needed for all diagnostic measures and for other previous comorbidity research. However, for some disorders, such as MDE and PD with or without agoraphobia, this revision should be none to very minor considering that the criteria changed very little or not at all. For other disorders, such as PTSD for which the criteria have undergone more changes, this revision may be more substantial. Therefore, comorbidity and the clusters that make up the various comorbidity structures will require reinvestigation as well, but we would expect little change for disorders for which the criteria have not changed considerably.

### Validation of Comorbidity Factors

The 4 comorbidity factor scores, anxiety-depression, substance abuse, body image-eating, and depression-sleep problems were related to several variables: suicidal ideation, adequate social support, quality of life, self-confidence, and the Kessler-6 total score. As expected, the regressions of all 6 dependent variables on the 4 comorbidity factors were statistically significant. The greater the comorbidity factor scores the greater the Kessler-6 total score (the greater the psychological distress). A search of various psychological databases revealed very little about the association between psychological distress and current comorbidity other than 1 study which found a positive association between them [68]. Another study that was more focused on adults with seizures also found a positive relationship between psychiatric and physical comorbidities and psychological distress [76]. It should be noted that most of the variation in the Kessler-6 total score was accounted for by the anxiety-depression factor, which is expected because of the direct relationship between Kessler-6 measures and measures of depression and anxiety.

As expected, the odds for having inadequate social support increased in likelihood for each additional point an individual scored on anxiety-depression, substance abuse, body image-eating, and depression-sleep problems factors, respectively. This is indeed expected because social relationships (involving social network composition, social support, social interaction frequency and quality, and the experience of isolation and loneliness) have long been linked with longevity and mental and physical health. Social support helps people to cope more adaptively with acute and chronic stress, thus potentially enhancing allostasis and autonomic nervous system (ANS) reactivity and related emotional regulation. Poor ANS reactivity and emotional dysregulation are generally considered core factors associated with many mental health conditions. Mazzella et al [71] concluded that mortality progressively increased with low social support and greater comorbidity, and that low social support progressively increased with increasing comorbidity in the elderly. Rockhill et al [72] found lack of social support mediated the association between symptoms and lower grades for adolescents with depression alone and comorbid symptoms. We did not find many studies that examined the direct relationship between social support and current comorbidity.

As expected, the odds for having low self-confidence to deal with one’s mental health issues increased in likelihood for each additional point an individual scored on anxiety-depression, substance abuse, body image–eating, and depression–sleep problems factors, respectively. These results suggest that greater comorbidity factor scores reduce self-confidence in one’s ability to deal with mental health issues. We are not aware of any study that directly examined the relationship between comorbidity of psychological disorders and patients’ perceived confidence in dealing with their mental health issues.

As expected, the odds for having poor quality of life increased in likelihood for each additional point an individual scored on anxiety-depression, substance abuse, body image-eating, and depression-sleep problems factors, respectively. Several studies have shown a reduced health-related quality of life for patients with physical comorbidities [76-79]. Fewer studies have also found a negative relationship between comorbidity and quality of life in specific populations, such as obese or bipolar patients [69,70]. But we are not aware of any study that directly examined psychological comorbidity and general quality of life. It should not be surprising to find a negative relationship between comorbidity factor scores and the general rating of one’s quality of life. The same mechanisms that result in reduced quality of life for patients with physical comorbidities should also produce reduced quality of life for those with psychological comorbidity.

As expected, the odds for experiencing suicidal ideation increased in likelihood for each additional point an individual scored on anxiety-depression, substance abuse, body image-eating, and depression-sleep problems factors, respectively. These results are particularly important and useful because they suggest that comorbidity factor scores on all scales have the potential to be used for the purpose of identifying those with suicidal ideation, especially for those with high scores on the anxiety-depression factor. However, more work is needed to validate the potential use of these factor scores. These results are consistent with the findings of Albert et al [3] and Schoevers et al [4] who argued that comorbidity was consistently related to higher rate of suicide.

There are very few studies published on the relationships between current psychological comorbidity and measures such as general psychological distress, self-confidence in dealing with one’s mental health, adequate social support, and quality of life. Future studies should further explore the relationships between these measures. The fact that we used only 1 simple question to measure these variables is a limitation of this study that places a constraint on the generalization of findings. A second limitation is that the e-PASS depends exclusively on automated self-report to assess and determine the diagnoses of participants. Andersson and Titov [80] raised some concerns about the use of an online assessment tool for the purpose of diagnosing individuals. Thirdly, there is only 1 study that examined the psychometric properties of the e-PASS (D Nguyen, unpublished PhD thesis, Swinburne University, 2013). More studies with larger samples and more established measures for comparisons based on the newly released *DSM-5* are needed before definitive conclusions can be made. A fourth limitation is that the 4 factors only managed to explain 44% of the total variance in the severity scores. This analysis needs to be repeated and a confirmatory factor analysis may be conducted on a new dataset collected since October 2012 to demonstrate the generalizability of this model.

## Abbreviations

ADIS: Anxiety Disorders Interview Schedule
ANS: autonomic nervous system
BDD: body dysmorphic disorder
DSM-5: Diagnostic and Statistical Manual of Mental Disorders (5th Edition)
DSM-IV-TR: Diagnostic and Statistical Manual of Mental Disorders (4th Edition, Text Revision)
e-PASS: electronic psychological assessment screening system
GAD: generalized anxiety disorder
KMO: Kaiser-Meyer-Olkin
MDD: major depressive disorder
MDE: major depressive episode
NCS: National Comorbidity Survey
OCD: obsessive-compulsive disorder
PCA: principal component analysis
PD: panic disorder
PTSD: posttraumatic stress disorder
SAD: social anxiety disorder
